# The effect of vitamin B_12_ supplementation in Nepalese infants on growth and development: study protocol for a randomized controlled trial

**DOI:** 10.1186/s13063-017-1937-0

**Published:** 2017-04-21

**Authors:** Tor A. Strand, Manjeswori Ulak, Ram K. Chandyo, Ingrid Kvestad, Mari Hysing, Merina Shrestha, Sudha Basnet, Suman Ranjitkar, Laxman Shrestha, Prakash S. Shrestha

**Affiliations:** 1 Department of Research, Innlandet Hospital Trust, 2629 Lillehammer, Norway; 2 Centre for International Health, University of Bergen, P.O. Box 7800, 5020 Bergen, Norway; 3 Department of Child Health, Institute of Medicine, Tribhuvan University, Maharajgunj, P.O. Box 1524, Kathmandu, Nepal; 4 Regional Center for Child and Youth Mental Health and Child Welfare West, Uni Research Health, Bergen, Norway

**Keywords:** Growth, Infants, Nutrition, Cobalamin, Nepal, Cognitive Development, Supplementation

## Abstract

**Background:**

Vitamin B_12_ deficiency is one of the most common micronutrient deficiencies and is associated with poor cognitive development and growth. Vitamin B_12_ is crucial for normal cell division and differentiation, and it is necessary for the development and myelination of the central nervous system. The aim of the present study is to measure the effect of daily supplementation of vitamin B_12_ on the neurodevelopment and growth of young children in Nepal.

**Methods/design:**

We are conducting an individually randomized, double-blind, placebo-controlled trial with 600 marginally stunted children 6–11 months old (length for age less than −1 z-score). Children are randomized to receive a lipid-based paste containing vitamin B_12_ or placebo daily for 12 months. The main outcomes are changes in growth (z-scores) and in neurodevelopment measured by the Bayley Scales of Infant and Toddler Development, Third Edition, from baseline until the end of the study.

**Discussion:**

If vitamin B_12_ supplementation benefits early child development and growth, this will have consequences for dietary recommendations for malnourished children worldwide.

**Trial registrations:**

ClinicalTrials.gov Identifier: NCT02272842. Registered on 21 October 2014.

Universal Trial Number: U1111-1161-5187. Registered on 8 September 2014.

**Electronic supplementary material:**

The online version of this article (doi:10.1186/s13063-017-1937-0) contains supplementary material, which is available to authorized users.

## Background

Micronutrient deficiencies represent a major challenge to child health in many low- and middle-income countries, and they may increase the risk of suboptimal development, impaired cognitive function, and poor growth [1]. Vitamin B_12_ (cobalamin) deficiency is among the most common micronutrient deficiencies [2, 3] because its main source is animal source foods, which are expensive and for cultural and religious reasons are often not eaten. Vitamin B_12_ deficiency is associated with impaired infant and child growth [4, 5].

In several studies of women and children, we have demonstrated that poor vitamin B_12_ status is common in South Asia [3, 6, 7]. Case studies have demonstrated harmful effects of severe vitamin B_12_ deficiency on the developing infant brain [8, 9]. The consequences of mild or subclinical vitamin B_12_ deficiency are less clear, but they have been shown to be associated with decreased cognitive performance in both elderly persons and children [10–13]. Researchers in three randomized controlled trials (RCTs) have measured the effect of vitamin B_12_ supplementation on neurodevelopment in children. In a Norwegian trial, an intramuscular injection of vitamin B_12_ substantially improved motor development in 6-week-old infants after 1 month [14]. Another trial with low-birth-weight children in Norway recently confirmed these findings [15]. We also found a beneficial effect of vitamin B_12_ supplementation for 6 months on neurodevelopment in young North Indian children [16]. The infants in these three studies had evidence of suboptimal vitamin B_12_ status, but none of the children had severe deficiency.

Vitamin B_12_ works with folate, and both are required for cell division, use of energy, and other important metabolic processes. Impaired cell growth is the main mechanism behind anemia in severe cobalamin or folate deficiency. Adequate cell growth is also important for the growing nervous system. Vitamin B_12_ deficiency may also have consequences through various metabolic pathways that can alter energy use, the production of neurotransmitters, and myelin [8]. Myelin is the principal component of white matter in the brain and is important for nerve conductivity. Disruptions in myelination alter the speed of conduction in multiple systems of the central and peripheral nervous systems [9].

The first 2 years of life is the most important period for brain growth and development of the nervous system [17]. The brain develops rapidly through neurogenesis, axonal and dendritic growth, synaptogenesis, myelination, and gliogenesis. These events happen at different times and build on each other [17]. Small impairments during critical periods can have serious consequences for the brain’s structural and functional capacity, with the possibility of persistence into adult life [18, 19].

In addition to growth and cognitive development outcomes, we will try to estimate long-term effects of vitamin B_12_ supplementation in the present study. As a secondary outcome, we have therefore included measurements of leukocyte telomere length as a biological marker of future health. Telomeres are the caps located at the ends of chromosomes that promote chromosomal stability [20]. The length of the telomere is an indication of remaining cell cycles of a particular cell line in the body, and it may serve as a biological clock to determine the life span of a cell and an organism [20]. The telomeres shorten by age, and shorter telomeres in the individual are a sign of a shortened life span [21, 22]. Stress may accelerate telomere shortening, and the length of telomeres is often considered to be a biomarker of chronic stress [23]. In children, telomere length has been linked to early adversity and psychological stress [24, 25], family violence [23, 26], lead exposure [27], and cigarette smoking [26], as well as zinc deficiency [28] and disturbances in the one carbon metabolism that occur during folate or B_12_ deficiency [29]. In a pilot study with Nepalese children, we estimated the relative telomere length in 66 infants and found that the plasma methylmalonic acid (MMA) concentration was inversely associated with the telomere length (unpublished results, personal communication T. Strand). This association, which persisted after adjusting for relevant confounders, suggests that poor vitamin B_12_ status in infancy results in shorter telomeres, possibly linking poor status in infancy to poorer health outcomes later in life. Whether vitamin B_12_ status causes shortening of telomeres needs to be confirmed using an experimental design such as the one in this trial.

Establishing a causal link between vitamin B_12_ deficiency and neurodevelopment must be done in a randomized, placebo-controlled trial that provides an adequate amount of vitamin B_12_ for a sufficiently long period. The two RCTs on Norwegian infants were undertaken in patient populations, and in the RCT done in North India, neurodevelopment was a secondary outcome. We designed the present RCT to measure the effect of daily supplementation of vitamin B_12_ for 1 year in marginally stunted children (length for age less than −1 z-score) on neurodevelopment and growth.

## Methods/design

### Study aim and design

The main aim of the study is to measure to what extent administration of 2 μg of vitamin B_12_ for 12 months improves neurodevelopment, growth, and hemoglobin (Hb) concentration in children aged 6–11 months at enrollment. The impact of the vitamin B_12_ supplementation will be measured using a randomized, double-blind, placebo-controlled study design. We will also measure the effect of supplementation on other outcomes important for child health and development.

### Specific aims of the study

The specific aims of the study are to measure, in infants 6–11 months of age in a population where poor vitamin B_12_ status and malnutrition is common, the following:

1. To what extent administration of 2 μg of vitamin B_12_ for 1 year improves developmental scores measured by the Bayley Scales of Infant and Toddler Development, Third Edition (Bayley-III), and the Ages and Stages Questionnaire, Third Edition (ASQ-3)
2. To what extent administration of 2 μg of vitamin B_12_ improves length-for-age, weight-for-age, and weight-for-length z-scores after 1 year, as well as growth velocity z-scores for the initial 6 months and the last 6 months of supplementation
3. To what extent administration of 2 μg of vitamin B_12_ for 12 months improves Hb concentration

### Secondary aims

Secondary aims of the study are the following:

4. Measure to what extent administration of 2 μg of vitamin B_12_ for 12 months leads to less telomere shortening
5. To identify subgroups for the above-mentioned outcomes that benefit from vitamin B_12_ administration
6. Measure to what extent vitamin B_12_ administration alters plasma cobalamin, MMA, and total homocysteine (tHcy) concentrations
7. In multiple regression analyses, identify predictors for early childhood development scores using variables reflecting socioeconomic status, early stimulation opportunities, morbidity, growth, micronutrient status, self-regulation, sleep, activity, and others

### Study design, setting, and population

**Fig. 1:**
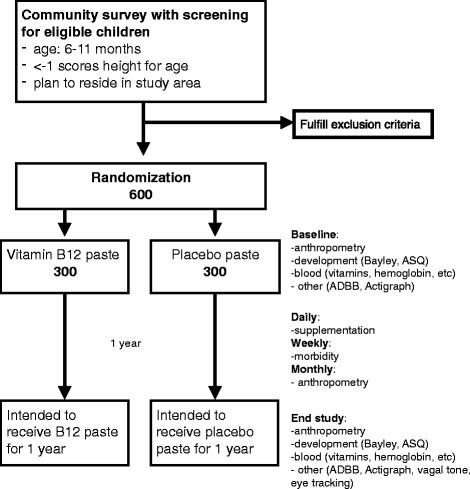
Flow of participants in a double-blind, randomized, placebo-controlled trial on the effect of daily supplementation of vitamin B_12_ on cognitive development, growth, and hemoglobin concentration. *ADBB* Alarm Distress Baby Scale, *ASQ-3* Ages & Stages Questionnaire, Third Edition, *Bayley* Bayley Scales of Infant and Toddler Development, Third Edition

This is a superiority, parallel group, community-based, individually randomized, double-blind, placebo-controlled trial. We are including 600 children aged 6–11 months with a length for age less than −1 z-score who plan to reside in the Bhaktapur municipality and surrounding areas in the district for the next 12 months and where caregivers are available for informed consent (Fig. 1). The study design follows the Standard Protocol Items: Recommendations for Interventional Trials (SPIRIT) guidelines (see Additional files 1 and 2).

### Procedures

#### Exclusion criteria

The following exclusion criteria will be applied:

1. Severe systemic illness requiring hospitalization
2. Severe malnutrition (i.e., weight for height less than −3 z-scores of the World Health Organization standard for this age group); for ethical reasons, these children require micronutrient supplementation and adequate medical care
3. Lack of consent
4. Taking B vitamin supplements that include vitamin B_12_
5. Severe anemia (Hb <7 g/dl); this would be a temporary exclusion, and the children will be enrolled if they are successfully treated
6. Ongoing acute infection such as fever or infection that requires medical treatment; this would be a temporary exclusion, and the children will be enrolled after recovery

#### Randomizing and blinding

Eligible children are allocated by block randomization (block sizes of 8) in a 1:1 ratio to one of the intervention groups. The vitamin B_12_ supplements and placebos have been produced specifically for this trial and are identical in taste and appearance. The supplements or placebos are offered in packages to the enrolled children according to a unique identification number provided by the company. The list that links the identification number to the randomization code is kept with the producers and with the scientist who generated the randomization code. None of the investigators have access to this list until the data collection and cleaning of data for the main outcomes are completed.

#### Intervention

All children receive a daily 2-μg oral supplement of vitamin B_12_ or placebo for 12 months. This amount of vitamin B_12_ for 12 months does not constitute any health risk for the child. However, we are closely monitoring possible adverse effects during follow-up.

The intervention is provided in sachets containing 20 g of lipid-based paste produced by GC Rieber Compact (Søfteland, Norway). Each sachet provides 1 day of supplements. The base paste for this study is the same as the eZeepaste® (GC Rieber Compact), which includes a multimicronutrient mixture (based on the United Nations Multiple Micronutrient Preparation formulation [30]). Thus, the placebo and vitamin B_12_ paste also contains several other vitamins and minerals at approximately 1 recommended dietary allowance. This is to ensure that the effect of vitamin B_12_ is not limited by deficiency of other important nutrients.

#### Cointervention

All caregivers are given dietary recommendations according to national guidelines. Children with anemia are given oral iron. At enrollment and the end of the study, we measure Hb in all children. Children with Hb <7 g/dl (exclusion criterion) at enrollment are treated with oral iron for 30 days and included in the study when their Hb rises to >7 g/dl. We measure Hb again in children who develop severe palmar pallor during follow-up. Children with Hb 7–10 g/dl are treated with peroral iron for 30 days (or longer if still anemic). Children with pneumonia, dysentery, or other illnesses are treated according to the Integrated Management of Childhood Illness guidelines [31]. All children with diarrhea receive 20 mg of zinc (dissolvable tablet, available locally) and oral rehydration solution.

#### Supervision of the daily follow-up

Supervisors organize the workflow from the central office. They also monitor the fieldworkers’ performance in the field by undertaking supervised and unsupervised visits to the field. In a random sample of the scheduled follow-ups, the supervisors follow the fieldworkers and observe their performance and interaction with the study participants. In the unsupervised visits, they visit the study participants after the fieldworkers have completed their tasks and ask questions about fieldworkers’ performance and behavior and, when possible, attempt to collect the same information as the fieldworkers. We undertake such supervised and unsupervised visits in 2% of all scheduled visits.

#### Ensuring compliance

During weekly visits in the homes, mothers are asked about intake of paste during the past 7 days. The amount of paste given to children is recorded in detail (i.e., half, one-third, three-fourths, or less). We also record the reason for the poor intake (i.e., child did not like the paste, mother forgot to give, or there was no paste left). All the occurrences of vomiting or regurgitation after supplementation of paste is recorded, and the total number of empty paste packets at the weekly visits is counted.

### Outcomes

#### Neurodevelopment

The neurodevelopmental assessments are carried out in designated rooms at the field clinic and in the homes of the children. The Bayley-III is the main neurodevelopmental outcome measure and is performed at baseline and the end of the study. The Bayley-III is a comprehensive assessment tool of developmental functioning in infants and toddlers aged 1–42 months [32]. The test is administered directly with the child and provides information on cognitive, language, and motor abilities. The Bayley-III represents the gold standard in assessment of this age group and is widely used for research purposes worldwide.

The ASQ-3 is an easily administered checklist of developmental status standardized for children aged 1–66 months. The screening system includes age-appropriate questionnaires for every 2-/ 3-month interval, and each questionnaire contains 30 items written in simple language. The questionnaires are designed to be completed by caregivers, but they can also be administered by a trained examiner such as in this project. The ASQ-3 has five subscales: communication, gross motor, fine motor, problem solving, and personal social. ASQ-3 is an easily administered and robust tool that we are using at baseline and the end of the study to compare the study groups.

The Alarm Distress Baby Scale (ADBB) is an instrument developed to measure social behavior and withdrawal in infants 2–24 months old (http://www.adbb.net/gb-echelle.html). The scoring is based on observation of the child, and the examination takes 10–15 minutes. Examiners have been trained and standardized to perform the ADBB observations according to existing standards of the ADBB program. The ADBB observations are video-recorded during enrollment and at the end of the study and scored by trained and validated examiners. We will compare the ADBB scores between the study groups at the end of the study. The ADBB scores will also be used in the multiple regression models to identify predictors for Bayley-III scores.

An actigraph (Actiwatch 2; Philips Respironics, Monroeville, PA, USA) is a wristwatch-like device that records motion data. Using a validated scoring algorithm, the Actiware® software (Philips Respironics) translates the activity data to sleep-wake patterns and activity levels. Sleep problems and sleep patterns are further corroborated by questionnaire-derived data (the Brief Infant Sleep Questionnaire [BISQ]; see below).

Heart rate variability (vagal tone) is a marker of parasympathetic activity. Vagal tone has been suggested to be a sensitive marker of self-regulation and cognitive control, and it may be an important early risk identifier of child development [33]. We are measuring vagal tone in children at the end of the study.

#### Questionnaires

The following questionnaires will be used:

1. The Home Observation for the Measurement of the Environment (HOME) is a structured interview and observational measure of the psychosocial environment and quality of maternal responsiveness/parental practice (http://www.safeguardingchildren.co.uk/appendix-5.html; North Yorkshire Safeguarding Children Board, Northallerton, UK). It has been used widely in international child development research and has been shown to be predictive of intellectual abilities. For the present study, we have selected several items relevant for the site for the assessment of the home environment.
2. The Caregiver Knowledge of Child Development Inventory (CKCDI) is a brief questionnaire used to assess parents’ knowledge of child developmental milestones and their knowledge of developmental stimulation [34]. Parental knowledge on child developmental milestones may be of importance to promoting adequate development in children. In this study, we are using the CKCDI when the children are 12 months old to account for the effect of the caregivers’ knowledge on child development.
3. The BISQ is a brief questionnaire on infant sleep that has demonstrated good psychometric properties as a sleep screening tool for clinical and research purposes in infants and toddlers (aged 0–30 months) [35].

#### Anthropometry

Weight is measured with a portable electronic scale that measures to the nearest 0.01 kg (Salter/HoMedics Group, Tonbridge, UK). Height and length are measured according to standard guidelines as we have done previously. Height and weight are measured at the clinic during enrollment and at the end of the study, as well as every month at the home visits during the 12-month supplementation period.

#### Clinical parameters

We obtain morbidity data on each child at our weekly visits. At the visits, mothers are asked about diarrheal illness (number and consistency of stools), symptoms of respiratory illness (cough, fast or difficult breathing), fever, and if there have been physician visits. Sick children who require treatment are referred to the study clinic for treatment.

#### Laboratory parameters

Blood samples are obtained at enrollment (baseline) and at the end of the 12-month follow-up period for each child. Three milliliters of blood is collected into vials containing ethylenediaminetetraacetic acid (EDTA) as an anticoagulant. The plasma is centrifuged at approximately 700 *g* at room temperature for 10 minutes, separated and transferred into storage vials, immediately transferred to liquid nitrogen in the field clinic, moved to another office in central Kathmandu, and stored at −80 °C. Dry ice is included when shipping the samples.

The blood samples will be/are analyzed for the following:

1. Hb
  - EDTA-preserved blood is analyzed for Hb by HemoCue B-Hemoglobin (HemoCue, Brea, CA, USA).
  - For children for whom blood from the cubital vein is not obtained, capillary blood is used for analysis of Hb.
2. Plasma vitamin B_12_ and plasma folate concentration will be estimated by microbiological assays using a chloramphenicol-resistant strain of *Lactobacillus casei* and colistin sulfate-resistant strain of *Lactobacillus leichmannii*, respectively [36, 37]. We will also measure plasma concentrations of MMA and tHcy, which are good markers vitamin B_12_ status.
3. Telomere length will be assessed using quantitative polymerase chain reactions [20] modified for a high-throughput 384-well format [38, 39]. Five nanograms of genomic DNA will be assayed in either the telomere or *36B4* (single-copy gene) polymerase chain reaction mixture. Laboratory technicians masked to participant characteristics will perform triplicate reactions of telomere and *36B4* reactions for each sample. The ratio of amount of the telomere amplification product (T) to that of a single-copy gene (S), or T/S ratio, for each sample will be calculated by subtracting the threshold cycle (C_t_) value for the average single-gene (*36B4*) copy number from the C_t_ value for the average telomere repeat copy number. The average relative T/S ratio will be calculated by subtracting the T/S ratio of a calibrator DNA sample from the T/S ratio of each sample (exponential 2^−ΔΔCt^). Quality control samples (10% of all samples) will be interspersed throughout each plate to assess interplate and intraplate variability.

### Training and quality control

Before the study start, the team arranged a training workshop in Nepal for the psychologists responsible for assessing children in the study. The psychologists and fieldworkers were trained in standardized use of the Bayley-III and the ASQ-3 for both the baseline and end-of-study assessments. A local psychologist and a local pediatrician served as “gold standards” both during training and throughout the study for the Bayley-III and the ASQ-3, respectively. Five percent of all sessions are scored by two examiners to ensure appropriate interobserver agreement. The standardization and quality control procedures are similar to the procedures used in our other ongoing studies. In addition, we videotape some of the Bayley-III assessments for checking by the supervising psychologists so that they can provide prompt feedback to the assessors. During the study period, there are weekly Skype (Skype Communications SARL, Luxembourg) meetings of the team to discuss progress in general and possible challenges that are faced. Additional training and standardizations are organized ahead of the end-of-study procedures.

### Sample size calculations

**Fig. 2:**
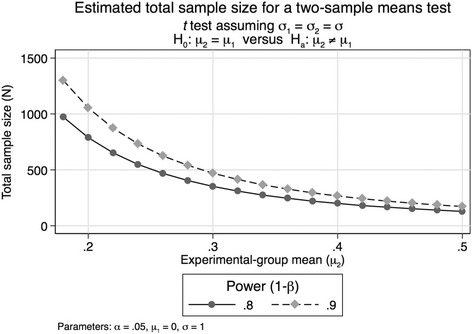
Required sample sizes by minimum meaningful effect sizes when comparing group means and assuming equal variances at 80% and 90% power

We have powered the study to have 90% power for most relevant outcomes, which are displayed in Fig. 2. The SD for Bayley-III, ASQ-3, and growth z-scores are derived from previous studies in Nepal and India and represent a worst-case scenario in terms of variability of the outcome measurements. The power to detect a difference of 4 Bayley-III points is almost 80% even with an SD of 17. The expected SD of the Bayley-III is normally 15. An effect size of 4 points corresponds to approximately 0.25 to 0.3 SD and is a commonly used figure in such trials. We would not expect a single intervention to have an effect beyond this magnitude. It should also be noted that we will compare the difference in cognitive scores from baseline to the end of the study between the groups, which will further reduce the variability and increase the statistical power.

### Data management and analysis

All forms are checked manually by supervisors for completeness and consistency. The data are then double-entered at the field clinic into appropriate databases with computerized range and consistency checks. In this process, there is continuous data cleaning. Comparison of baseline features, including vitamin B_12_ status, will be made to confirm comparability across treatment groups. Most of the data is entered into a Microsoft Access database (Microsoft, Redmond, WA, USA), but we are also using the iFormBuilder system (Zerion Software, Herndon, VA, USA) to enter the data into tablets. A separate database has been made for personal information such as names, addresses, pictures of homes, and global positioning system (“GPS”) coordinates. Only the principal investigators have access to this information after it has been entered.

We will use the Stata (StataCorp, College Station, TX, USA) and R (R Foundation for Statistical Computing, Vienna, Austria) statistical software packages to analyze the data. A detailed plan for analysis of the main outcomes will be made before the list that links the child identity numbers to the treatment groups is obtained by the researchers. Data cleaning, definition of outcome variables, exclusion of cases, and programming of scripts in the statistical packages will also be done before the analysis files are merged with the randomization lists. The intervention groups will be coded so that the researchers do not know the group identities until the main analyses are finished. In these analyses, there will be one variable denoting the group identity.

The analyses will be planned and undertaken in a joint workshop attended by the involved scientists. All analyses will initially be done on an intention-to-treat-basis. To adjust for potential baseline differences, we will use multiple regression models. All randomized participants will be included in the analyses if the relevant outcome variables have been collected. The main outcomes are continuous and expected to be normally distributed. We will use the change in Bayley-III scores from baseline to the end of the study in separate analyses where B_12_ supplementation is the main exposure. We will compare the mean Bayley-III scores (total score and scores on the subscales: cognitive, language, and motor, with the motor scale analyzed both separately for fine and gross motor development and as a composite measure) between the vitamin B_12_ group and the placebo group. A fully specified statistical analysis plan will be provided before opening the database and breaking the randomization code.

For the secondary outcomes, we will use a variety of statistical approaches. There is a range of possible analyses where the data may be used as predictors, mediators, or moderators. Before such analyses commence, we will lay out a plan of analysis for each of the research questions that will be addressed.

We will analyze the effect of vitamin B_12_ separately in the following subgroups:

1. Vitamin B_12_ status
  - Based on cobalamin (cutoff 150 pmol/L)
  - Based on low tHcy (cutoff 10 μmol/L)
  - Based on low MMA (cutoff 0.26 μmol/L)
2. Maternal body mass index (cutoff 18.5 kg/m^2^)
3. Vegetarian (yes/no)
4. Birthweight (cutoff 2500 g)

### Per-protocol analysis

In addition to a standard per-protocol analysis, we will use instrumental variable analysis in an attempt to estimate the true effect of cobalamin had it been given to all children in the scheduled doses and intervals. The random allocation will be the instrument in these analyses. For per-protocol analysis, women who receive <50% of the projected doses will not be included in the analyses while acknowledging that the ensuing effect estimates not only may be biased but also will certainly represent an effect higher than what can be achieved even in our well-resourced study setting.

### Publication

The results derived from this study will be published in international peer-reviewed biomedical journals. We will also inform policy makers such as national health authorities and the World Health Organization regarding our findings if relevant.

### Data and Safety Monitoring Board

The Data and Safety Monitoring Board (DSMB) is comprised of a pediatrician, a public health expert, and a biostatistician. The DSMB is independent from the sponsor and has no competing interests. The DSMB members review severe adverse events every 6 months. After one-third of the study participants were enrolled and completed follow-up, the DSMBs reviewed the data for serious side effects. No specific stopping rules were employed.

## Discussion

This proposed project is designed to measure the effect of vitamin B_12_ on neurodevelopment, growth, and Hb concentration in 600 Nepalese young children. We undertake the study within the critical window for brain development, start after other foods (in addition to breast milk) are introduced to the child, and end before the child reaches 24 months of age. In this Nepalese population, stunting is common, and, in contrast to the children that we have studied in North India [3, 40], folate deficiency is virtually nonexistent [6, 7]. The duration of vitamin B_12_ administration will be twice as long as the duration of supplementation in the study in India, and the children are younger [40]. As a result, there is a greater potential for catchup growth in the present study sample. Thus, we believe that the proposed study is well designed for measuring the effect of vitamin B_12_ on neurodevelopmental outcomes, growth, and Hb concentration.

If we are able to demonstrate a causal relationship between vitamin B_12_ and neurodevelopment and growth in malnourished children in low-income countries, this may influence national and international nutrition recommendations. Demonstrating that vitamin B_12_ is important for neurodevelopment and/or growth can ensure that vitamin B_12_ is included in micronutrient formulations that are recommended or available to vulnerable populations. If vitamin B_12_ is an important growth- and development-limiting factor, supplementation of this single nutrient has the potential to improve growth and development in many children.

We enrolled the first child in April 2015. One week after the first enrollment, the field site was severely affected by major earthquakes. Because of these catastrophes, we stopped the enrollment for 1 month, and our staff was used for relief work while following the first five enrolled children. After approximately 1 month, we were able to resume study enrollment and at the same time provide health services to the population of Bhaktapur. In September of the same year, Nepal was struck by a different disaster as a consequence of an embargo of goods from India. During a 6-month period, the supply of imported goods, including petrol, cooking gas, and medicines, was critically low. This severe situation also affected our ongoing project. However, because we were using predominantly local staff and because our consumables were in place before the study started, we were able to maintain enrollment and follow-up during this period also. As a result of these incidents, the study period will continue for longer than initially planned. According to the current enrollment rate, we enrolled the last child in March 2017, with follow-up 1 year later in March 2018.

## Trial status

As of April 2017, we had enrolled all 600 children; 25 had been lost to follow-up, mainly because of migration after the earthquake in Nepal; and 320 children had completed supplementation and the end of study activities.

## Additional files

Additional file 1: SPIRIT 2013 checklist: recommended items to address in a clinical trial protocol and related documents. (DOC 123 kb)

Additional file 2: Randomized trial on daily Vitamin B_12_ supplementation in Nepali infants: main activities (enrollment, interventions, and assessments). (PDF 403 kb)

## Abbreviations

ADBB: Alarm Distress Baby Scale
ASQ-3: Ages & Stages Questionnaire, Third Edition
Bayley-III: Bayley Scales of Infant and Toddler Development, Third Edition
BISQ: Brief Infant Sleep Questionnaire
CKCDI: Caregiver Knowledge of Child Development Inventory
C_t_: Threshold cycle
DSMB: Data and Safety Monitoring Board
EDTA: Ethylenediaminetetraacetic acid
GPS: Global positioning system
Hb: Hemoglobin
HOME: Home Observation for the Measurement of the Environment
MMA: Methylmalonic acid
RCT: Randomized controlled trial
SPIRIT: Standard Protocol Items: Recommendations for Interventional Trials
tHcy: Total homocysteine
T/S ratio: Ratio of amount of the telomere amplification product (T) to that of a single-copy gene (S)
