# Supplementary material for: The effect of vitamin B12 supplementation in Nepalese infants on growth and development: study protocol for a randomized controlled trial
Source: Trials. 2017 Apr 21;18:187. doi: 10.1186/s13063-017-1937-0 (PMC5399862; doi:10.1186/s13063-017-1937-0)
Supplement: Supplementary file 2 — Randomized trial on daily Vitamin B12 supplementation in Nepali infants: main activities (enrollment, interventions, and assessments). (PDF 403 kb) [file 13063_2017_1937_MOESM2_ESM.pdf]

Additional file 2 Randomized trial on Daily Vitamin B12 Supplementation in Nepali infants: Main activities (enrolment, interventions, and assessments). *

	**STUDY PERIOD**							
	**Enrolment**	**Allocation**	**Every month for the first year**					**Close-out**
**TIMEPOINT****	***$-t_1$***	**0**	***t_1***	***t_2***	***t_3***	***t_4***	***etc.***	***t_x***
**ENROLMENT:**								
**Eligibility screen**	X							
**Informed consent**	X							
Anthropometry Cognitive development]	X							
**Allocation**		X						
**INTERVENTIONS:**								
multivitamins - vitamin B12			—	—	—	—	—	
multivitamins + vitamin B12			—	—	—	—	—	
**ASSESSMENTS:**								
Socioeconomic, demographic	X							
Cognitive development	X							X
Monthly Anthropometry	X		X	X	X	X	X	X
Weekly Morbidity			X	X	X	X	X	X
Biochemical (vitamin status)	X							X
Diet / feeding	X							
Home environment					X			
Neurophysiological tests								X

*Assessments: Cognitive: Bayley Scales of Infant Development version 3 and Ages and Stages Questionnaire -3, Anthropometry: length and weight.

**Every month or every week throughout the first year
